# Assessment of economic development of central business districts – A combination multi-criteria evaluation methodology

**DOI:** 10.1371/journal.pone.0326877

**Published:** 2025-07-31

**Authors:** Kaiyuan He, Bingbing Huang, Xinyu Liu, Shouzhen Zeng

**Affiliations:** 1 School of Business, Ningbo University, Ningbo, China; 2 Economic Forecasting and Policy Simulation Laboratory, Zhejiang Gongshang University, Hangzhou, China; 3 School of Business and Research Academy of Belt and Road, Ningbo University, Ningbo, China; University of Economics Ho Chi Minh City, VIET NAM

## Abstract

**Background:**

The economic development of central business districts (CBDs) is a key driver of urban economic growth and enhances regional competitiveness. However, existing research predominantly focuses on macro-level analyses, lacking systematic and quantitative indicator systems for detailed evaluation of CBD economic development performance, while studies on urban office buildings or specific CBD components often neglect the broader context of the entire district.

**Objectives:**

This study aims to propose a multi-criteria evaluation system to effectively evaluate the economic development performance of CBDs, and provide theoretical references for a more comprehensive evaluation of the sustainable CBD growth in other regions.

**Methods:**

Firstly, the analytic hierarchy process (AHP) and entropy method are proposed to determine combination weights for each indicator. The VlseKriterijumska Optimizacija Kompromisno Resenje (VIKOR) method is then introduced to rank the economic development performance of CBDs. Finally, a numerical example of Zhejiang province is carried out to validate the results by comparing its rankings with existing methods.

**Results and conclusion:**

The multi-criteria evaluation of regional CBD economic development showed that the top two cities remained consistent across subjective, objective, and combined weights methods, while discrepancies in rankings for other cities were balanced by the combined weights approach. Meanwhile, minor variations in rankings highlighted the unique advantages of each method. These findings validate the effectiveness of combining subjective and objective methods for assessments. Future research should enhance data comprehensiveness and reliability through fieldwork to further improve the scientific validity of such evaluations.

## 1. Introduction

The concept of Central Business Districts (CBDs) was first introduced by Burgess through his concentric zone model, which divided urban areas into five zones based on their social functions. At the core lies the CBD, serving as the focal point for retail activities, business offices, service provision, and other tertiary-sector activities [1]. This model illustrates a spatial structure where the CBD forms the innermost zone, radiating outward in a pattern of decreasing economic intensity.

**Fig 1:**
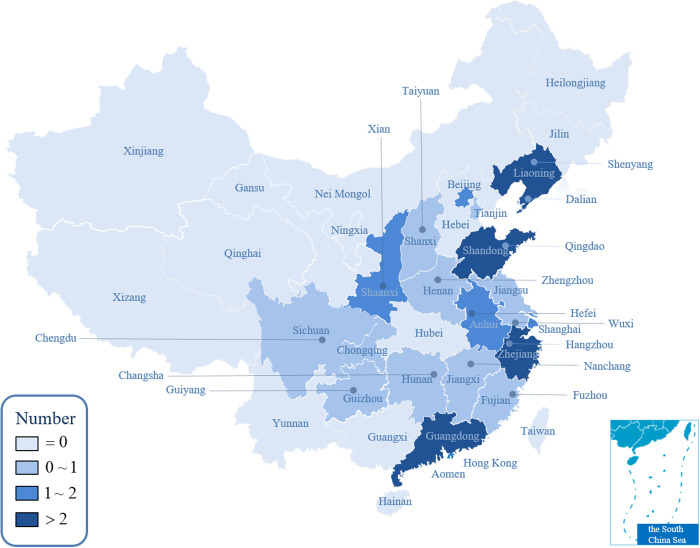
Top 30 benchmark CBD in China.

**Fig 2:**
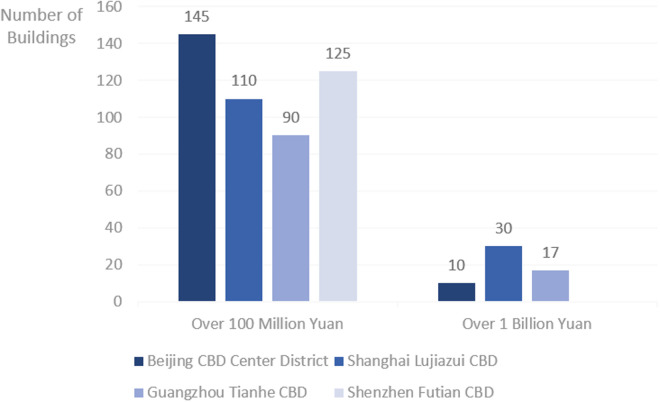
Tax revenue distribution of buildings in major CBDs (2023). Note: Data on buildings generating tax revenues exceeding 1 billion yuan in Shenzhen Futian CBD is unavailable and therefore excluded from this chart.

The emergence of CBDs in Chinese cities can be traced back to the 1980s, driven by the country’s economic reforms and an open-door policy [2]. This period saw the rapid growth of the real estate market alongside China’s transition from a manufacturing-based economy to a service-oriented model, leading to significant spatial reconfigurations in urban landscapes [3,4]. Cities such as Shanghai and Shenzhen were among the first to experience the economic transformation of their CBDs, which became hubs of modern business activities. By the 1990s, local governments began prioritizing the development of “urban new districts”, integrating industrial parks and office areas as part of a broader strategy for urban expansion [5]. As a central hub for urban planning and decision-making, the CBD represents one of the most dynamic and prominent urban spaces in modern Chinese cities [6,7]. Recent data from the National Bureau of Statistics of China highlights the rapid expansion of CBD-related infrastructure: in 2023, real estate investment in office buildings reached 453.078 billion yuan, with 20 major cities contributing approximately 6 million square meters of new office space—an increase of 19.6% year-on-year. The 2024 China CBD economic development index identifies the top 30 benchmark districts, with a majority concentrated in eastern provinces and first-tier cities, as illustrated in Fig 1. The CBDs in cities such as Beijing, Shanghai, Guangzhou, and Shenzhen exhibit outstanding performance, generating substantial local tax revenues and setting a benchmark for CBD development nationwide. Fig 2 illustrates the distribution of buildings generating tax revenues exceeding 100 million yuan and 1 billion yuan in 2023 across four of China’s most representative CBDs.

In Western countries, factors such as locational advantages and market dynamics are primary determinants of CBD success [8,9]. In China, the development of CBDs has been largely shaped by state-led initiatives, underscoring the significant role of government restructuring and policy interventions [10]. Additionally, globalization and economic shifts have played a crucial role in influencing CBD growth patterns, further differentiating regional dynamics [11,12]. Despite these differing growth drivers, CBDs consistently function as the economic and social nucleus of cities, assuming a pivotal strategic role in an increasingly globalized world. Their development not only tackles key challenges, including limited urban development space but also enhances the industrial structure of cities, improves both the employment landscape and business environment. Moreover, CBDs serve as a driving force for sustainable socio-economic development by fostering innovation, economic diversification, and urban competitiveness [2]. In contemporary societies, they act as strategic nodes within global urban networks, hosting advanced producer services and supporting flexible, decentralized production systems critical for maintaining global competitiveness [13].

However, the challenges faced by CBDs have become increasingly complex in the context of changing development priorities and external pressures. Traditionally centered on location, rent, and hardware facilities, the development of CBDs has gradually shifted toward emphasizing sustainability, environmental quality, and social benefits alongside economic efficiency. This balancing act remains a formidable challenge, as CBDs are under pressure to simultaneously adapt to demands for industrial upgrading, technological innovation, and diversified economic structures. The challenges are further exacerbated by the disruptive impact of the COVID-19 pandemic, which has fundamentally altered traditional patterns of urban activity. The proliferation of telework has diminished the centrality of office spaces and weakened the traditional agglomerative advantages of CBDs [14,15]. As competition between CBDs intensifies, the need for more refined, specialized, and diversified approaches to development has become increasingly urgent, highlighting the necessity of a comprehensive evaluation of CBDs’ economic development in a rapidly changing world.

Scholarly research to address these challenges has primarily focused on improving architectural design, optimizing spatial organization, and strengthening policy guidance to achieve better environmental sustainability, operational efficiency, and adaptability to changing urban demands. For instance, Yaguang [16] emphasized the need for sustainable design principles to mitigate the adverse environmental and social impacts of dense office infrastructure, thereby fostering healthier and more livable urban environments. Huang et al. [17] provided insights into how climate zones, urbanization rate, building electrification, and population influence carbon emissions. Shi et al. [18] pointed out that indoor settings optimizing economic benefits align with those maximizing productivity, emphasizing the need to prioritize worker productivity in air conditioning design. Säynäjoki et al. [19] demonstrated how sustainability-oriented architectural designs could enhance environmental performance, while Barbhuiya et al. [20] emphasized how green building certifications and circular economy approaches can drive market transformation and promote sustainable development in the construction industry. In spatial organization of CBDs, Taubenböck et al. [21] analyzed the spatial distribution and patterns of CBDs in megacities, contributing to an understanding of their functional integration within urban systems. Yu et al. [6], Murphy and Vance [22] investigated CBD land utilization and delineation through advanced spatial techniques, such as network kernel density estimation, to better define their boundaries and configurations. Gao et al. [23] identified the distance-decaying trends in shopping district vitality using mobile phone data, underscoring the importance of land area and industrial agglomeration in shaping CBD performance. Pan [24] examined the proximity between CBDs and surrounding employment centers, revealing its significant impact on productivity spillovers and advocating for a reconsideration of traditional urban equilibrium models. Moreover, Gibson et al. [14] explored decision-making frameworks to propose alternative strategies for revitalizing CBDs in ways that align with broader urban development goals.

These studies not only provide meaningful perspectives on environmental sustainability and spatial optimization of CBDs but also lay a theoretical and practical foundation for promoting their sustainable economic development. However, more targeted evaluation methods and indicator systems for assessing the economic development performance of CBDs remain lacking, which are crucial for identifying their strengths, weaknesses, and potential for sustainable growth. Existing evaluation indicator systems for CBDs, often focus on either narrowly assessing internal economic activities which lack comprehensiveness or adopting overly broad frameworks that lack quantifiable metrics. This limitation restricts their capacity to inform data-driven decision-making and effectively guide the strategic development of CBDs. To address these limitations, this study proposes a scientifically evaluation indicator system for CBD economic development, structured around three core dimensions: innovation capability, urban economic development level, and urban infrastructure and public services. The detailed construction of the indicator system will be discussed in the next section. The key highlights of this study are as follows:

(1) Development of a comprehensive evaluation indicator system for CBDs: This study constructs an evaluation indicator system that captures the economic development performance of CBDs while serving as a practical tool for policy making. By integrating multidimensional indicators, the indicator system helps stakeholders assess strengths and weaknesses, prioritize objectives, and align CBD strategies with regional and sustainability goals. Furthermore, it improves theoretical understanding by providing approach to analyzing complex systems and unifying diverse indicators within a coherent analytical structure.
(2) Combination of analytic hierarchy process (AHP) and entropy weight method (EWM) for indicator weighting: To ensure methodological rigor, this study combines the AHP and the EWM for determining indicator weights, effectively balancing subjective expert judgment with objective data analysis. This approach enhances the scientific precision and rationality of the weighting process, addressing the limitations of purely subjective or objective methods often found in existing studies.
(3) Application of VIKOR for comprehensive evaluation: The evaluation system employs VlseKriterijumska Optimizacija Kompromisno Resenje (VIKOR) as the primary method to assess the economic performance of CBDs, focusing on ranking alternatives by balancing trade-offs among conflicting criteria. To ensure methodological rigor and validate the results, the TOPSIS method is applied as a secondary tool for comparative analysis. The complementary use of VIKOR and TOPSIS enhances the robustness of the evaluation process by leveraging the strengths of each method. This methodological synergy strengthens the reliability and comprehensiveness of the assessment.
(4) Case study application in Zhejiang Province: This study applies the proposed evaluation system to a case study in Zhejiang, demonstrating its practical relevance in assessing the economic development performance of CBDs. The case study offers empirical insights into how the indicator system adapts to regional characteristics and varying urban dynamics. Based on the findings, this research proposes targeted policy recommendations to enhance CBD economic development.

## 2. CBD evaluation index system

The evaluation index systems for CBDs are typically constructed around key dimensions such as infrastructure, economic development, market competitiveness, and the human environment. Xu [25] identified six primary categories for constructing an evaluation index system for CBDs in China. These categories encompass: regional economic development indicators, basic condition indicators, business environment indicators, market openness indicators, humanistic environment indicators, and other competitiveness indicators. He et al. [26] proposed a five-dimensional evaluation framework encompassing “urban innovation capacity, economic development level, government support, industrial structure, and infrastructure level” in their study on the impact of smart city pilot initiatives on urban innovation mechanisms. This framework effectively assesses the economic and innovation performance of urban areas.

At the specific indicator level, Wang et al. [27] constructed an inter-regional science and technology innovation capability evaluation index system from the aspects of environment, input, output, efficiency and performance to solve the problem of large gaps in economic development levels caused by unbalanced development of science and technology innovation capabilities among regions. Işık et al. [28] proposed an evaluation framework based on factors such as economic performance, research and development, cultural interaction, livability, environmental conditions, and accessibility. This framework, used to compare the competitiveness of major European cities. Lu et al. [29] established a comprehensive evaluation framework for assessing the development level of a green, low-carbon, and circular economy, focusing on socio-economic benefits such as per capita disposable income. Chang proposed a set of indicators for measuring and assessing CBD economic activity to support planning and decision-making. These indicators encompass essential metrics across land use and economic dimensions, such as employment and productivity, as well as transportation efficiency, communication networks, and social or human security [30]. Yu et al. [31] explored the impact of land-based fiscal reliance on urban sprawl and spatial planning in China, proposing indicators such as local government fiscal revenue, per capita GDP, total retail sales of consumer goods, and fixed asset investment to evaluate urban development dynamics. Xu et al. incorporated [32] indicators such as fixed asset investment to reflect the ecological efficiency issues in urban sustainable development.

Based on the above analysis, this paper establishes an indicator system to evaluate the current performance of CBD economic development, including three dimensions: innovation capability (micro-level competitiveness), urban economic development level (meso-level potential), and urban infrastructure and public services (macro-level support). The system also takes into account the rationality of indicator selection and data availability. This indicator system integrates the core driving factors of CBD economic growth, enabling a more comprehensive evaluation. Table 1 provides an overview of this indicator system, which includes 17 specific indicators.

**Table 1:** Indicator system for CBD economic development evaluation.

First-Level Index	Second-Level Index	Indicator unit	Source (Ref.)
Innovation capability (A)	Total R&D expenditure (A_1_)	Hundred million yuan	[27,28]
	Number of patent applications granted (A_2_)	Applications	[27,28]
	Number of employees in the tertiary industry (A_3_)	Thousands of people	[27,28]
Urban economic development level (B)	Per capita GDP (B_1_)	Yuan	[27]
	Per capita disposable income of urban residents (B_2_)	Yuan	[29]
	Consumer price index (B_3_)	–	[28]
	Gross industrial output value (B_4_)	Hundred million yuan	[30]
	Proportion of tertiary industry GDP to regional GDP (B_5_)	Percent	[27]
	Gross output value of tertiary industry (B_6_)	Hundred million yuan	[27]
	Total retail sales of consumer goods (B_7_)	Hundred million yuan	[28,31]
	Retail price index (B_8_)	–	[28]
Urban infrastructure and public services (C)	Total fiscal revenue (C_1_)	Hundred million yuan	[31]
	General public budget expenditure (C_2_)	Hundred million yuan	[31]
	Investment in real estate development (C_3_)	Hundred million yuan	[31]
	Increase in fixed asset investment (C_4_)	Percent	[31,32]
	Highway mileage per city (C_5_)	Kilometer	[28,30]
	Number of office buildings (C_6_)	Building	

The meanings of the indexes are examined in detail below:

(1) **Innovation ability (A).** Innovation capacity highlights the competitiveness of local enterprises and their ability to foster long-term growth, contributing to social advancement. The selected indicators include:
  Total R&D expenditure (A_1_): This metric quantifies regional expense in research and development, reflecting the commitment to fostering high-tech industries and advancing innovation-driven growth.
  Number of patent applications granted (A_2_): Serving as a key indicator of technological vitality, this metric highlights the region’s capacity for competitiveness in intellectual property creation.
  Number of employees in the tertiary industry (A_3_): This indicator assesses the workforce engaged in the tertiary sector, a critical driver of CBD economic development, reflecting the sector’s scale and capacity to support innovation-led activities.
(2) **Urban economic development level (B).** Urban economic development level evaluates the economic vitality and development potential of the region, representing regional economic benefits. This dimension is assessed through the following indicators:
  Per capita GDP (B_1_): A direct and authoritative measure of a region’s overall economic performance.
  Per capita disposable income of urban residents (B_2_): Reflects the economic activity and purchasing power of residents, indicating the overall standard of living.
  Consumer price index (B_3_): The changes in the prices of goods and services, offering insights into economic stability and the cost of living, which impact CBD economic development.
  Gross industrial output value (B_4_): Captures the total economic output of the industrial sector, highlighting production efficiency and its role in supporting CBD economic growth.
  Proportion of tertiary industry GDP to regional GDP (B_5_): Measures the service sector’s contribution to the urban economy, indicating modernization and sustainability potential.
  Gross output value of tertiary industry (B_6_): Assesses the overall economic scale of the service sector, encompassing activities such as finance, education, and retail, to gauge market activity and economic health.
  Total retail sales of consumer goods (B_7_): Reflects the consumption demand and market activity, showcasing residents’ income levels and spending capacity.
  Retail price index (B_8_): Evaluates fluctuations in commodity prices, influencing consumer behavior and corporate strategies, with indirect effects on CBD economic performance.
(3) **Urban infrastructure and public services (C).** Urban infrastructure and public services capture the foundational support necessary for sustaining CBD operations and attractiveness, promoting environmental benefits through efficient resource utilization and sustainable urban systems. This dimension is evaluated through the following indicators:
  Total fiscal revenue (C_1_): Reflects the city’s overall financial capacity and its ability to allocate resources for infrastructure and public services, which are critical to CBD development.
  General public budget expenditure (C_2_): Represents government spending on urban infrastructure and public services, which directly determines their availability, quality, and ability to meet the demands of CBD development.
  Investment in real estate development (C_3_): Captures the scale of real estate projects, which contribute to the physical environment and supporting facilities essential for attracting investors and promoting CBD expansion.
  Increase in fixed asset investment (C_4_): Measures the level of infrastructure investment, which stimulates economic growth and job creation, reflecting economic development trends.
  Highway mileage per city (C_5_): Represents the accessibility and connectivity of urban areas, influencing resource flow, spatial structure, and overall operational efficiency within the city.
  Number of office buildings (C_6_): Serves as a direct measure of CBD activity and scale, reflecting the economic vitality and business environment of the region.

## 3. The integrated weights based on AHP and EWM

### 3.1. Subjective weight based on AHP

The AHP method, introduced by Saaty [33], has been extensively utilized as a robust method for deriving normalized weights for multi-criteria decision-making problems [34–36]. By decomposing complex decision-making tasks into hierarchical structures, AHP facilitates systematic evaluations of criteria at various levels [37]. Central to this method is the pairwise comparison of criteria, where subjective judgments from decision-makers are quantified to determine relative priorities. The weighting system is established by calculating the eigenvector corresponding to the largest eigenvalue of the pairwise comparison matrix, ensuring consistency and validity in the rankings. The traditional AHP method involves the following steps:

**Step 1.** Constructing the judgment matrix: *A* pairwise comparison matrix is developed to represent the relative importance of various criteria. The elements of the matrix are determined using a predefined numerical scale (ranging from 1 to 9), reflecting the comparative preference of one criterion over another.


$$A=\left[\begin{array}{cccc} {}*20cC_{11} & C_{12} & ... & C_{1n}\\ C_{21} & C_{22} & ... & C_{2n}\\ ... & ... & ... & ...\\ C_{n1} & C_{n2} & ... & C_{nn} \end{array}\right]$$ (1)

**Step 2.** Normalizing the matrix: Each column of the judgment matrix is normalized by dividing each element by the column sum.

**Step 3.** Calculating relative weights: The priority vector is obtained by averaging the normalized values in each row, representing the relative importance of each criterion within the hierarchy.

**Step 4.** Consistency verification: To ensure logical coherence in the pairwise comparisons, the consistency index (CI) and consistency ratio (CR) are computed as follows:


CR=CI/RI (2)

$$CI=(\lambda_{\max}-n)/(n-1)$$ (3)

where $\lambda_{\max}$ is the maximum eigenvalue of the judgment matrix, n is the matrix dimension, and RI represents the random consistency index determined by the size of the matrix. CR values below 0.1 indicate acceptable consistency, while higher values necessitate a revision of the matrix to improve reliability.

Finally, the relative importance of all factors with respect to the overall objective is calculated, and their rankings are validated through consistency testing.

### 3.2. Objective weight based on EWM

The EWM, rooted in Shannon’s information entropy theory [38], quantifies the relative intensity of indicators based on their variability and interactions. This method has found extensive applications across diverse domains, including engineering, regional planning and environmental studies [39–42]. By leveraging entropy to measure the degree of dispersion within indicator data, the EWM assigns weights objectively. In this context, higher entropy values signify smaller variations between attribute values, thereby indicating reduced information content. The steps for determining the weights of objective indicators using the EWM are outlined as follows:

**Step 1.** Construct the index matrix:

Let $R={\left(r_{ij}\right)}_{m\times n}$ represents the index matrix, where i=1,2,3,...,m corresponds to the alternatives, and j=1,2,3,...,n corresponds to the evaluation criteria. The matrix entries $r_{ij}$ denote the value of the j -th criterion for the i -th alternative.


$$R=\left[\begin{array}{ccc} {}*20cr_{11} & ... & r_{1n}\\ ... & ... & ...\\ r_{m1} & ... & r_{mn} \end{array}\right]$$ (4)

**Step 2.** Normalize the index matrix:

To eliminate the influence of scale differences among indicators, the matrix is standardized as follows:


$$x_{ij}=\frac{r_{ij}-\max\{r_{ij}\}}{\max\{r_{ij}\}-\min\{r_{ij}\}}$$ (5)

**Step 3.** Calculate the entropy of each criterion:


$$b_{j}=-\frac{1}{\ln m}\sum_{i=1}^{m}a_{ij}\ln a_{ij},i=1,2,3,...,m;j=1,2,3,...,n$$ (6)

where


$$a_{ij}=\frac{x_{ij}}{\sum_{i=1}^{m}x_{ij}},i=1,2,3,...,m;j=1,2,3,...,n$$ (7)

**Step 4.** Determine the entropy weight:


$$w_{j}^{EWM}=\frac{1-b_{j}}{n-\sum_{j=1}^{n}b_{j}},j=1,2,3,...,n$$ (8)

**Step 5.** Objective weight vector of the index:


$$W_{j}^{EWM}=(w_{1}^{EWM},w_{2}^{EWM},...,w_{n}^{EWM})$$ (9)

### 3.3. Integrated weights

The AHP method derives indicator weights based on expert judgment, capturing their qualitative assessment of the relative importance of criteria. While this approach reflects domain-specific expertise, its reliance on subjective evaluations introduces a potential lack of objectivity. Conversely, the EWM calculates weights based on the inherent variability of data, ensuring a quantitative and unbiased weighting process. However, solely relying on objective methods may result in discrepancies, as the calculated weights might fail to align with practical realities or contextual nuances, leading to deviations in the research outcomes.

To overcome the inherent limitations of subjective and objective weighting approaches, this study proposes a combination of subjective (AHP) and objective (EWM) methods. This combined methodology aims to mitigate the shortcomings of both methods and enhance the scientific and rational nature of decision-making [43]. The integrated AHP-EWM approach is widely recognized for its capacity to derive comprehensive weights that balance subjective insights with objective data analysis, ensuring a more scientific and reliable evaluation of indicators. A higher weight signifies greater relevance to the evaluation criteria, reinforcing the importance of accurate weight determination in multi-criteria decision-making. For instance, Li et al. [44] employed the AHP-EWM in assessing wetland degradation risks, calculating weights to identify degradation trends and their underlying causes. Huang et al. [45] utilized AHP-EWM to prioritize disaster relief demand indicators, facilitating the allocation of resources based on the urgency of needs across affected regions.

**Fig 3:**
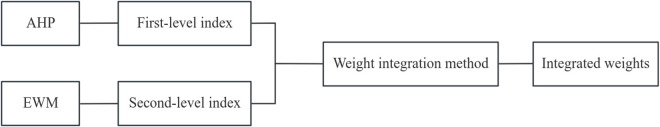
Flowchart of weight integrated calculation.

The subjective weights of the first-level indicators obtained by AHP and the objective weights of the second-level indicators obtained by EWM are integrated by linear weighting method to calculate the relative importance of the final second-level indicators. The calculation steps are illustrated in Fig 3.

The detailed process is as follows:

**Step 1.** Construct subjective and objective weight vectors:

The subjective weight vector obtained by AHP:


$$W_{j}^{AHP}=(w_{1}^{AHP},w_{2}^{AHP},...,w_{n}^{AHP})$$ (10)

and the objective weight vector obtained by EWM:


$$W_{j}^{EWM}=(w_{1}^{EWM},w_{2}^{EWM},...,w_{n}^{EWM})$$ (11)

**Step 2.** For index j, calculate the product of its subjective and objective weights:


$$w_{j}^{AHP}\times w_{j}^{EWM}$$ (12)

**Step 3.** Sum the products and get the normalized denominator S:


$$S=\sum_{j=1}^{n}\left(w_{j}^{AHP}\times w_{j}^{EWM}\right)$$ (13)

**Step 4.** Calculate the normalization weight of each index by Eq. (14):


$$W_{j}=\frac{{w_{j}}^{AHP}\times w_{j}^{EWM}}{S}$$ (14)

## 4. Evaluation methods

The VIKOR method is a widely utilized comprehensive evaluation approach designed for optimizing decisions in complex systems across various fields [46]. Its appeal lies in its simplicity, operational efficiency, cost-effectiveness, and ability to deliver accurate results. By ranking alternative solutions based on their proximity to positive and negative ideal solutions, the VIKOR method facilitates the selection of optimal decision-making strategies. This compromise-based approach is particularly well-suited for resolving conflicts among multiple criteria, as it identifies solutions that achieve a balanced trade-off between competing objectives. The practical utility of the VIKOR method is demonstrated across diverse domains. For instance, Wu et al. [47] applied the VIKOR method to compare solutions aimed at enhancing bank efficiency, ranking banks based on attitude indices. Similarly, Abdul et al. [48] employed VIKOR to rank weights derived from the AHP method, facilitating the selection of appropriate renewable energy alternatives for Pakistan. Furthermore, Meniz et al. [49] introduced an extended VIKOR model integrating Interval Type-2 Fuzzy Sets (IT2FS) to address vaccine selection challenges during the COVID-19 pandemic.

The VIKOR method utilizes an aggregation function derived from the $L_{p}$ -metric, expressed as follows:


$$L_{pj}=\left\{\sum_{i=1}^{n}\left[w_{i}\left(f_{i}\;\;*-f_{ij}\right)/\left(f_{i}\;\;*-f_{i}^{-}\right)\right]p\right\}\frac{1}{p}$$ (15)

Here, j represents the number of influencing factors; and i denotes the total number of evaluation criteria; $f_{ij}$ corresponds to the i -th evaluation value of the j -th influencing factor; $f_{i}^{*}$ and $f_{i}^{-}$ represent the positive ideal solution and the negative ideal solution, respectively; p is the distance parameter used in the aggregation function (set to 1 in this study); n denotes the total number of criteria; $w_{i}$ indicates the weight assigned to the i -th criterion, and $L_{pj}$ represents the aggregated distance of the j -th alternative to the ideal solution.

The VIKOR method comprises the following steps:

**Step 1.** Normalize the data by the Eq. (16):


$$x_{ij}=\frac{r_{ij}}{\sqrt{\sum_{i=1}^{m}r_{ij}^{2}}},\;i=1,2,3,...,m;j=1,2,3,...,n$$ (16)

**Step 2.** Determine the positive ideal solution ($f_{i}^{*}$) and the negative ideal solution ($f_{i}^{-}$), i=1,2,3,...,m.

**Step 3.** Calculate the integrated distance $S_{i}$ between each alternative and the ideal solutions, as well as the distance $R_{i}$ to the most unfavorable criterion, using the following relations:


$$\begin{array}{c} S_{i}=\sum_{i=1}^{m}w_{j}\left(f_{j}^{*}-f_{ij}\right)/\left(f_{j}^{*}-f_{j}^{-}\right)\\ R_{i}=\underset{j}{\max}\left[w_{j}\left(f_{j}^{*}-f_{ij}\right)/\left(f_{j}^{*}-f_{j}^{-}\right)\right] \end{array}$$ (17)

where $w_{j}$ denotes the weight of the j -th criteria, reflecting its relative importance.

**Step 4.** Calculate the compromise indicator $Q_{i}$ (i=1,2,3,...,m), which integrates both $S_{i}$ and $R_{i}$, using Eq. (18):


$$Q_{i}=v\frac{\left(S_{i}-S^{*}\right)}{\left(S^{-}-S^{*}\right)}+\left(1-v\right)\frac{\left(R_{i}-R^{*}\right)}{\left(R^{-}-R^{*}\right)}$$ (18)

where v is the weight coefficient representing the decision-maker’s preference for the majority criterion (0<v < 1); $S^{*}$ and $S^{-}$ are the minimum and maximum $S_{i}$ values; $R^{*}$ and $R^{-}$ are the minimum and maximum $R_{i}$ values.

**Step 5.** Rank all alternatives based on their calculated $S_{i}$, $R_{i}$ and $Q_{i}$ values in ascending order. The alternative with the smallest $Q_{i}$ is identified as the compromise solution. If multiple alternatives have the same $Q_{i}$, they are considered potential compromise solutions.

## 5. Case study and comparative analysis

### 5.1. Case study

As a coastal economic powerhouse in China, Zhejiang Province serves as a representative region in China for investigating CBD development, given its dynamic economy and high level of urbanization. Data from 2023 shows that in Zhejiang Province, Hangzhou had 246 buildings with tax revenues exceeding 100 million yuan, including 20 buildings generating over 1 billion yuan. Yinzhou District in Ningbo recorded 30 buildings with tax revenues exceeding 100 million yuan. However, both cities show a significant gap compared to first-tier cities such as Beijing, Shanghai, Guangzhou and Shenzhen.

This study evaluates the economic development of CBDs across 11 prefecture-level cities in Zhejiang Province, based on the indicator system outlined in Section 2. The data used in this analysis was sourced from the Zhejiang Statistical Yearbook. The detailed process is described below.

**Step 1.** Experts were invited to score the indicators according to the standardized tiers of the indicator system. To ensure the reliability of subjective weighting, 12 experts from fields such as urban economics, sustainable development, transportation planning, and real estate economics were carefully selected to participate in the evaluation process. These fields align closely with the multidimensional nature of CBD development, covering economic, social, and spatial perspectives. To address potential discrepancies in expert opinions, individual scores were compared to the group average. The two experts whose evaluations deviated most significantly from the consensus were excluded. Subsequently, the scores from the remaining 10 experts were averaged to calculate the final indicator weights. According to Eqs. (1)–(3), the weight values of the criteria layer obtained through the AHP method are $W_{0}^{\left(A\right)}$ = [0.14726 0.52402 0.32872]. Then, the weights of the index layer were subsequently calculated:

$W_{1}^{\left(A\right)}$ = [0.45767 0.41601 0.12632]
$W_{2}^{\left(A\right)}$ = [0.24325 0.17898 0.05316 0.09362 0.17912 0.15472 0.07042 0.02664]
$W_{3}^{\left(A\right)}$ = [0.28710 0.33185 0.15498 0.09578 0.08385 0.04644]

Here, $W_{0}^{\left(A\right)}$ represents the criteria layer’s weights, while $W_{1}^{\left(A\right)}$, $W_{2}^{\left(A\right)}$, $W_{3}^{\left(A\right)}$ are the index layer’s weights. The judgment matrix passed the consistency test, confirming that the weights are logically consistent and reasonable.

**Step 2.** Using data from the Zhejiang Statistical Yearbook, the values for each indicator across different cities are shown in S1 Appendix. The data were subsequently normalized, and the EWM was applied to compute the objective weights of the secondary indicators, following the calculations showed in Eqs. (4)–(9). The calculated weights are presented in Table 2.

**Table 2:** Indicator weights based on EWM.

Item	Information entropy	Information utility value	Weight coefficient
A_1_	0.7886	0.2114	7.47%
A_2_	0.8526	0.1474	5.21%
A_3_	0.868	0.132	4.66%
B_1_	0.8573	0.1427	5.04%
B_2_	0.9307	0.0693	2.45%
B_3_	0.8821	0.1179	4.16%
B_4_	0.8495	0.1505	5.32%
B_5_	0.865	0.135	4.77%
B_6_	0.7879	0.2121	7.49%
B_7_	0.8497	0.1503	5.31%
B_8_	0.8761	0.1239	4.37%
C_1_	0.73	0.27	9.54%
C_2_	0.8654	0.1346	4.76%
C_3_	0.8188	0.1812	6.40%
C_4_	0.8966	0.1034	3.65%
C_5_	0.9457	0.0543	1.92%
C_6_	0.5048	0.4952	17.49%

**Step 3.** The composite weights for the indicators were determined using Eqs. (10)–(14). Table 3 presents the results, including the subjective weights, objective weights, and the combined weights for all indicators.

**Table 3:** Combined weights of indicators.

Criterion layer	Index level	AHP weights	EWM weights	Combined weights
Innovation ability A	A_1_	6.74%	7.47%	8.91%
	A_2_	6.13%	5.21%	5.65%
	A_3_	1.86%	4.66%	1.53%
Urban economic development level B	B_1_	12.75%	5.04%	11.37%
	B_2_	9.38%	2.45%	4.07%
	B_3_	2.79%	4.16%	2.05%
	B_4_	4.91%	5.32%	4.62%
	B_5_	9.39%	4.77%	7.93%
	B_6_	8.11%	7.49%	10.75%
	B_7_	3.69%	5.31%	3.47%
	B_8_	1.40%	4.37%	1.08%
Innovation ability C	C_1_	9.44%	9.54%	15.93%
	C_2_	10.91%	4.76%	9.19%
	C_3_	5.09%	6.40%	5.77%
	C_4_	3.15%	3.65%	2.03%
	C_5_	2.76%	1.92%	0.94%
	C_6_	1.53%	17.49%	4.72%

As shown in Table 3, Total R&D expenditure (A_1_), Per capita GDP (B_1_), Gross output value of tertiary industry (B_6_), Total fiscal revenue (C_1_), and General public budget expenditure (C_2_) exhibit relatively higher weight values, signifying their substantial influence on the evaluation of CBD economic development. In contrast, indicators such as the Number of employees in the tertiary industry (A_3_), Retail price index (B_8_), and Highway mileage per city (C_5_) have comparatively lower weight values, reflecting a marginal impact on the assessment of CBD economic development performance.

**Step 4.** The current state of CBD economic development in Zhejiang was evaluated using the VIKOR method, with v = 0.5. The calculations were performed based on Eqs. (16)–(18). The evaluation results are presented in Table 4.

**Table 4:** Evaluation Results of CBD economic development in Zhejiang Province.

Cities	S	R	Q	Ranking
Hangzhou	0.06	0.0184	0	1
Ningbo	0.324	0.0551	0.2875	2
Wenzhou	0.6756	0.1357	0.7831	3
Jiaxing	0.727	0.1315	0.799	4
Huzhou	0.8017	0.1446	0.8901	8
Shaoxing	0.6961	0.1392	0.8078	5
Jinhua	0.754	0.1408	0.8479	6
Quzhou	0.8803	0.1593	0.9889	10
Zhoushan	0.841	0.1545	0.9485	9
Taizhou	0.7804	0.1427	0.8706	7
Lishui	0.8988	0.1592	0.9997	11

As shown in Table 4, significant variations exist in the performance of CBD economic development across cities in Zhejiang Province. The top three cities: Hangzhou, Ningbo, and Wenzhou, demonstrate relatively strong comprehensive performance in CBD economic development. Notably, Hangzhou and Ningbo exhibit clear advantages in their overall scores, while Wenzhou and Jiaxing display relatively similar scores. At the lower end of the ranking, Quzhou and Lishui show weaker performance, highlighting a noticeable gap in CBD economic development compared to other cities in the province.

### 5.2. Comparison of subjective and objective weighting

**Fig 4:**
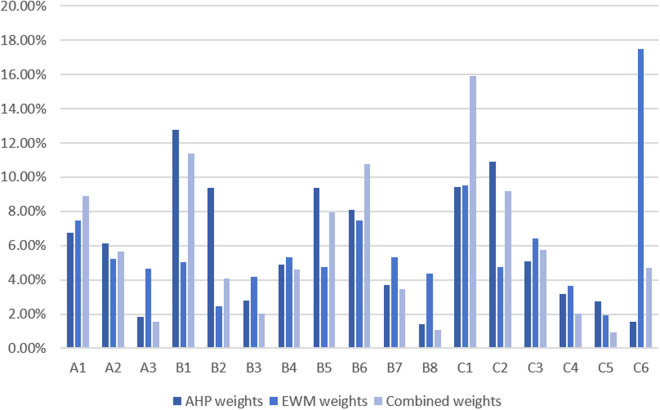
Comparison of indicator weights under different methods.

The AHP method leverages expert experience and judgment to assign weights to each indicator, effectively capturing the experts’ intuitive perception of their relative importance. In contrast, the EWM derives weights through data-driven analysis, reflecting the actual significance of each indicator based on the underlying data. These two methods adopt distinct criteria for weight determination, resulting in differing weight distributions. Fig 4 compares the indicator weights obtained through the different weight calculation methods.

**Fig 5:**
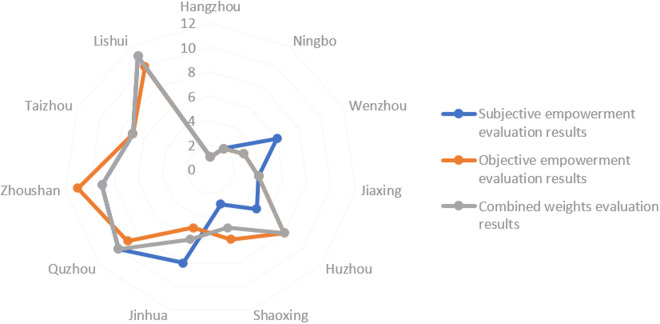
Comparison of rankings under different weighting methods.

In this section, the weights obtained from AHP and EWM are applied separately to rank the cities using the VIKOR method, enabling a comparison of the strengths and limitations of these two weighting approaches. The ranking results are presented in Tables 5 and 6, while Fig 5 visualizes the comparison of these results.

**Table 5:** Evaluation results based on AHP weights.

Cities	S	R	Q	Ranking
Hangzhou	0.076	0.0269	0	1
Ningbo	0.2985	0.0652	0.3325	2
Wenzhou	0.6134	0.1137	0.7744	6
Jiaxing	0.6789	0.0934	0.7152	4
Huzhou	0.7443	0.095	0.7651	5
Shaoxing	0.6288	0.0842	0.6376	3
Jinhua	0.6929	0.1203	0.8582	8
Quzhou	0.8384	0.1075	0.8874	10
Zhoushan	0.7864	0.1091	0.8619	9
Taizhou	0.7285	0.1035	0.7973	7
Lishui	0.8595	0.1275	1	11

**Table 6:** Evaluation results based on EWM.

Cities	S	R	Q	Ranking
Hangzhou	0.0942	0.035	0	1
Ningbo	0.3647	0.1043	0.4156	2
Wenzhou	0.7008	0.1639	0.8369	3
Jiaxing	0.7361	0.1711	0.8845	4
Huzhou	0.7981	0.1728	0.929	8
Shaoxing	0.7497	0.1735	0.9014	6
Jinhua	0.7501	0.1716	0.8951	5
Quzhou	0.8431	0.1744	0.9625	9
Zhoushan	0.9006	0.1749	1	11
Taizhou	0.8034	0.1714	0.9273	7
Lishui	0.89	0.1748	0.993	10

When comparing the rankings generated by the combined weighting method with those under the subjective assignment approach, significant differences can be observed. Specifically, under the subjective weighting method, Wenzhou and Jinhua experience a marked decline in their rankings, while Huzhou and Shaoxing show notable improvements. This discrepancy arises from the inherent limitations of subjective weighting, which is influenced by expert bias and subjective judgment. For instance, the indicators Per capita disposable income of urban residents (B_2_) and Highway mileage per city (C_5_) are assigned relatively high weights, while Total fiscal revenue (C_1_) and Number of office buildings (C_6_) are assigned comparatively low weights. The imbalanced weighting reveals the role of subjective judgment in shaping the rankings.

Under the objective weighting method, the rankings display different trends. Jinhua and Shaoxing swap positions, with Jinhua advancing to fifth and Shaoxing falling to sixth, while Zhoushan drops significantly from ninth to eleventh place. This reflects the sensitivity of the EWM to variations in the dataset. Outliers or fluctuations in the data can result in pronounced shifts in rankings, which compromises the stability and reliability of the results. Additionally, the EWM may overlook the intrinsic relationships between indicators, as it emphasizes mathematical consistency.

In multi-indicator decision-making methods, the ranking may be affected to some extent when an indicator is excluded or given extreme weights. This study adopts the combination of AHP and entropy weighting method, which theoretically provides an effective buffer mechanism. On the one hand, AHP assigns subjective weights through expert judgment, which can dynamically adjust the weight distribution when the indicator structure changes; on the other hand, entropy weight method automatically adjusts objective weights based on the data distribution characteristics, which can reflect the dynamic changes in the importance of indicators due to the increase or decrease of information. The combination of the two forms a comprehensive weight, which helps to reduce the impact of single weight fluctuations on the overall ranking under the scenario of indicator changes. Although rankings may fluctuate as a result of changes in certain key indicators, such changes are effectively moderated within the model, reducing the risk of systematic skewing of rankings.

By integrating subjective and objective weighting methods, the combined weighting strategy effectively balances the empirical judgment of experts with the objectivity of data-driven analysis. This approach not only mitigates the potential biases inherent in individual methods but also enables a more holistic and accurate assessment of the CBD economic development performance of each city.

### 5.3. Comparison with TOPSIS method

The TOPSIS is a widely used ranking method designed to approximate the ideal solution by simultaneously considering the positive and negative ideal solutions within an evaluation framework [50]. This method determines the degree of variation by calculating the Euclidean distances between each evaluation object and the ideal solutions. One of the primary advantages of TOPSIS lies in its minimal reliance on subjective input from decision-makers, making it a robust and versatile tool for decision-making across various domains [51–54].

The TOPSIS and VIKOR differ significantly in their approach to addressing multi-criteria decision-making problems, particularly in their normalization processes and decision focus. TOPSIS employs vector normalization to remove units from the criterion functions, while VIKOR utilizes linear normalization [55]. Furthermore, VIKOR aims to achieve a balance between maximizing the overall group benefit and minimizing individual regret, making it particularly effective in resolving conflicting objectives and identifying compromise solutions. TOPSIS offers greater stability by focusing on the proximity of alternatives to the ideal solution, while it exhibits inherent limitations. Specifically, it assumes static ideal and negative ideal solutions, neglects potential correlations between candidate solutions, and prioritizes proximity to the ideal solution without considering other critical factors influencing comprehensive decision-making.

**Fig 6:**
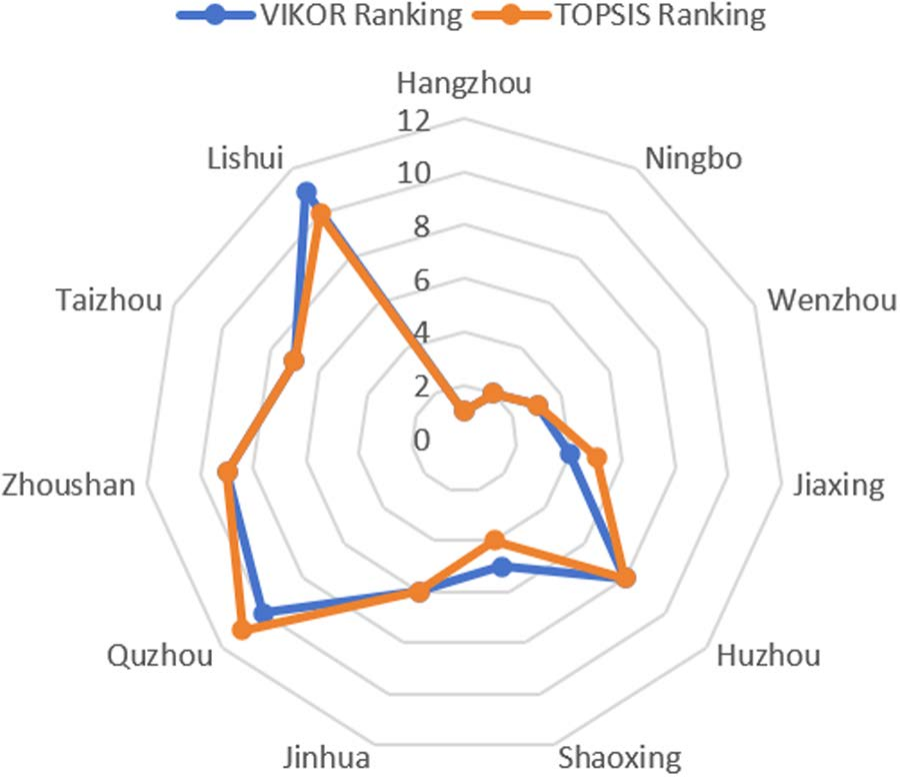
Rankings under different evaluation methods.

To facilitate a comparative analysis of CBD economic development across cities in Zhejiang Province, we applied the TOPSIS method. By calculating the relative distances of each city from the positive and negative ideal solutions, we derived their relative proximity scores, which served as the basis for ranking the cities. The results are presented in Table 7, with a visual comparison of the rankings generated by the VIKOR and TOPSIS methods illustrated in Fig 6.

**Table 7:** Evaluation Results of TOPSIS.

Cities	D	D-	C	Ranking
Hangzhou	0.06	0.577	0.905	1
Ningbo	0.23	0.381	0.623	2
Wenzhou	0.433	0.187	0.302	3
Jiaxing	0.469	0.139	0.228	5
Huzhou	0.523	0.079	0.13	8
Shaoxing	0.47	0.143	0.233	4
Jinhua	0.485	0.13	0.211	6
Quzhou	0.571	0.064	0.1	11
Zhoushan	0.577	0.082	0.124	9
Taizhou	0.489	0.116	0.192	7
Lishui	0.567	0.072	0.112	10

Comparing the results in Table 7 with the rankings derived from the VIKOR, it can be seen that there is a high degree of consistency in the overall evaluation of the CBD’s economic development by the cities, with the top three ranking cities remaining consistent. However, there are still four cities that produced changes in their rankings, with Shaoxing and Lishui moving up one place each under the TOPSIS, while Jiaxing and Quzhou dropped one place.

Hangzhou, Ningbo and Wenzhou are consistently ranked in the top two under both VIKOR and TOPSIS, attributed to their balanced lead in indicators with higher combination weights (C_1_, B_1_, B_6_, C_2_, A_1_). For VIKOR, this multi-indicator advantage is synchronized and amplified under the dual constraints of weighted distance S and maximum regret R, ultimately presenting a low Q. In TOPSIS, the distance between the original values of these cities and the positive ideal solution is extremely small, keeping their relative closeness C high, and the ranking logics of the two methods converge when a city is at the top of several core indicators.

The core differences between VIKOR and TOPSIS lie in their approaches to handling multi-criteria conflicts and extreme values. VIKOR, by balancing “group benefit” and “individual regret,” exhibits a natural smoothing effect on extreme values through the adjustment of the compromise coefficient v and its rankings place greater emphasis on the overall balance of performance. For instance, in this study, Jiaxing’s relatively balanced overall performance resulted in a higher ranking under the VIKOR method. In contrast, TOPSIS emphasizes the proximity of alternatives to positive and negative ideal solutions, using Euclidean distance to highlight the impact of single high-weight or high-performance indicators. As a result, Shaoxing, with its prominent tertiary industry output (B_6_), achieves a higher ranking under the TOPSIS method. The mathematical logic and decision-making priorities of the two methods differ fundamentally, directly leading to discrepancies in rankings for certain cities.

The ranking differences reflect the sensitivity of the methods to data characteristics. Under TOPSIS’s weighting mechanism, the advantage of a single indicator tends to be amplified, whereas VIKOR’s compromise mechanism requires stronger overall performance across multiple criteria, reducing its reliance on any single dominant indicator. This logical divergence suggests that TOPSIS is more suited to identifying cities with significant contributions from specific indicators, but it may overlook the overall balance of data. In contrast, VIKOR seeks to achieve equilibrium among conflicting indicators. The observed ranking differences not only reveal the applicability characteristics of the two methods but also highlight the importance of multi-method validation. The combined application of VIKOR and TOPSIS can mitigate potential ranking biases caused by reliance on a single method, thereby enhancing the comprehensiveness and robustness of the evaluation. In practice, the adjustment of VIKOR’s compromise coefficient v could be explored further to improve the model’s resistance to data fluctuations and extreme values, ensuring the stability and reliability of evaluation results across different data environments.

## 6. Conclusions

This study developed a comprehensive index system to evaluate the performance of regional CBD economic development, focusing on three key dimensions: innovation capability, urban economic development level, and urban infrastructure and public services. The VIKOR method was used for evaluation, and the indicator weights were determined in combination with AHP-EWM, combining the subjective judgment of experts with objective data analysis. A comparative analysis of subjective and objective assignments reveals that for cities with large differences in evaluation results, the evaluation results of the comprehensive weighting method are more balanced. In addition, a comparison using the TOPSIS method reveals that the top three cities remain consistent, further validating the stability of the method.

The results of the study highlight the effectiveness of the combined subjective and objective weighting method in realizing the assessment, providing a replicable indicator system for evaluating the economic development of CBDs, and at the same time having the potential to be widely applied to solve the problem of calculating and evaluating the weights of multiple indicators. In cross-regional applications, it can be used to enhance adaptability by introducing region-specific indicators, adjusting the weight calculation system, and optimizing the model by combining expert opinions and local policies in response to the differences in economic structure, data availability and institutional environment in different regions. In addition, the methodology has important promotion value in the fields of industrial cluster planning and assessment, regional economic performance analysis, and infrastructure project assessment.

This study shows that the sustainable development of CBD needs to balance economic growth and resource utilization efficiency, while strengthening technological innovation to meet the current challenges of uneven resource allocation, inefficient industrial clustering, and insufficient innovation capacity. To this end, targeted strategies can be adopted in the following three areas:

(1) Promote balanced development: Prioritize the construction of infrastructure and public services in lower-ranked urban CBDs to reduce regional disparities and achieve equitable economic growth.
(2) Optimize resource allocation: Enhance the efficiency of resource integration through the establishment of resource-sharing mechanisms (e.g., office space, technology platforms, and business services) to promote industrial collaboration within CBDs.
(3) Promote the construction of innovation clusters: Deepen cooperation among enterprises, universities and the government to develop emerging areas such as the digital economy and green industries, and build competitive innovation clusters to strengthen the long-term sustainability of the CBD.

## Supporting information

S1 Appendix Table A. Indicator data of each city in Zhejiang. (DOCX)
