# Supplementary material for: Assessment of economic development of central business districts – A combination multi-criteria evaluation methodology
Source: PLoS One. 2025 Jul 31;20(7):e0326877. doi: 10.1371/journal.pone.0326877 (PMC12312973; doi:10.1371/journal.pone.0326877)
Supplement: S1 Appendix — (DOCX) [file pone.0326877.s001.docx]

**Appendix A.**

**Table A.** Indicator data of each city in Zhejiang

|  | Hangzhou | Ningbo | Wenzhou | Jiaxing | Huzhou | Shaoxing | Jinhua | Quzhou | Zhoushan | Taizhou | Lishui |
| --- | --- | --- | --- | --- | --- | --- | --- | --- | --- | --- | --- |
| A1 | 723.03 | 460.96 | 209.4 | 232.41 | 126.89 | 221.01 | 136.65 | 45.25 | 40.91 | 155.58 | 39.9 |
| A2 | 121196 | 76068 | 55500 | 39085 | 18954 | 33181 | 43198 | 7059 | 2741 | 36555 | 10363 |
| A3 | 465.19 | 273.76 | 270.75 | 144.77 | 87.94 | 150.13 | 207.37 | 58.13 | 31.94 | 189.63 | 76.39 |
| B1 | 151527 | 163280 | 82961 | 121410 | 112804 | 137325 | 78048 | 87486 | 166777 | 90457 | 72798 |
| B2 | 77043 | 76690 | 73326 | 72096 | 71044 | 76199 | 69626 | 57465 | 71965 | 70737 | 55784 |
| B3 | 102.4 | 102.3 | 101.8 | 102.1 | 102.3 | 101.7 | 101.9 | 102.5 | 101.7 | 101.6 | 101.9 |
| B4 | 4922 | 6682 | 2706 | 3379 | 1772 | 3030 | 2021 | 716 | 840 | 2239 | 566 |
| B5 | 68.18 | 50.36 | 55.68 | 42.67 | 44.74 | 47.74 | 55.23 | 51.72 | 42.53 | 50.85 | 55.02 |
| B6 | 12787 | 7909 | 4471 | 2876 | 1723 | 3509 | 3072 | 1036 | 830 | 3072 | 1007 |
| B7 | 7293.6 | 4896.7 | 3944.1 | 2342.7 | 1594.7 | 2585.9 | 2965.3 | 878.1 | 575.2 | 2586 | 804.8 |
| B8 | 102.9 | 104.1 | 102.7 | 103.8 | 103.7 | 102.9 | 103.3 | 103.5 | 103.3 | 102.8 | 102.6 |
| C1 | 4590.08 | 3358.63 | 917.1 | 1029.99 | 674.5 | 821.64 | 779.41 | 277.88 | 407.64 | 726.54 | 280.29 |
| C2 | 2004.25 | 1520.99 | 1187.58 | 579.31 | 490.04 | 662.58 | 842.87 | 441.39 | 266.02 | 680.52 | 628.1 |
| C3 | 3889.1 | 2131.68 | 1540.67 | 1038.66 | 704.07 | 1067.89 | 788.41 | 265.57 | 197.86 | 972.36 | 343.25 |
| C4 | 6 | 10.4 | 7.8 | 7.2 | 9.1 | 11 | 13.5 | 17.7 | 4 | 6.5 | 16 |
| C5 | 16632 | 11470 | 15533 | 8246 | 8222 | 10480 | 13232 | 8735 | 1935 | 13298 | 15135 |
| C6 | 3038 | 1240 | 213 | 89 | 60 | 48 | 80 | 33 | 24 | 84 | 26 |
